# Importance of species traits on individual-based seed dispersal networks and dispersal distance for endangered trees in a fragmented forest

**DOI:** 10.3389/fpls.2022.1010352

**Published:** 2022-09-21

**Authors:** Ning Li, Xifu Yang, Yuanhao Ren, Zheng Wang

**Affiliations:** ^1^ Institute of Applied Ecology, Nanjing Xiaozhuang University, Nanjing, China; ^2^ State Key Laboratory of Integrated Management of Pest Insects and Rodents in Agriculture, Institute of Zoology, Chinese Academy of Sciences, Beijing, China; ^3^ College of Biology and Environmental Science, Nanjing Forestry University, Nanjing, China

**Keywords:** individual-based seed dispersal network, bird foraging type, plant individuals, *Taxus chinensis*, habitat fragmentation

## Abstract

Although mutualistic network analyses have sparked a renewed interest in the patterns and drivers of network structures within communities, few studies have explored structural patterns within populations. In an endangered tree species population, plant individuals share their bird seed dispersers; however, the factors affecting individual interaction patterns are poorly understood. In this study, four individual-based networks were built for the endangered Chinese yew, *Taxus chinensis*, in a fragmented forest based on bird foraging type (swallowing and pecking networks) and habitat type (networks in a bamboo patch and an evergreen broad-leaved forest patch). Species-level network metrics (species degree and specialization, *d’*) were used to evaluate the effects of species traits (bird and plant traits) on species-level networks and dispersal distance for *T. chinensis*. It was revealed that the interaction networks between *T. chinensis* individuals and their bird partners were influenced by foraging type and the habitat of plant distribution. Compared to the other two networks, bird swallowing and bird–fruit networks in the evergreen broad-leaved patch habitat had higher nestedness and connectance but lower modules and specialization. Bird (body weight and wing and bill lengths) and plant traits (height, crop size, and cover) significantly affected species-level network metrics such as degree and specialization. Furthermore, seed dispersal distance was influenced by species traits and the species-level metrics of fruit–bird interaction networks. These results provide new insights into individual-based seed dispersal mutualistic networks of endangered plant species under habitat fragmentation. Moreover, these findings have relevant implications for conserving and managing individual endangered trees in increasingly disturbed ecosystems.

## Introduction

Habitat fragmentation is one of the most important drivers of the decline in species diversity (Dirzo et al., 2014; Haddad et al., 2015; Cao and Zhang, 2022), which may trigger cascading effects on ecological networks (Taubert et al., 2018; Peters et al., 2019). In most remnant habitat patches, mutualistic networks for animal-mediated pollination and seed dispersal are disrupted by species loss (Schupp et al., 2017; Grass et al., 2018) and affected by the size and quality of habitat patches (Spiesman and Inouye, 2013; de Bomfim et al., 2018; Emer et al., 2019). Compared with large habitat patches, small habitat patches are unable to support the survival of most large-bodied species owing to their lower food quality and greater interspecific competition (Hart et al., 2017; Ferretti and Fattorini, 2021). Consequently, the seed dispersal networks dominated by these large-bodied species are disrupted, thus affecting subsequent plant species recruitment (Bregman et al., 2016; Donoso et al., 2016).

Species naturally interact to form complex networks, which have different structural characteristics such as nestedness (Almeida-Neto et al., 2008; Krishna et al., 2008), connectance (Dunne et al., 2002), modularity (Olesen et al., 2007), specialization (Bluthgen et al., 2006; Schleuning et al., 2011), and species degree (Bascompte et al., 2006). The structural characteristics of these networks provide ecologists with a better understanding of the mechanism underlying the interactions among species (Bascompte and Jordano, 2007). In recent years, the topological structure of ecological networks has been well-developed at the community and species levels (Bluthgen et al., 2006; Thebault and Fontaine, 2010; Montesinos-Navarro et al., 2017; Yang et al., 2018). However, studies on these network structures have largely ignored potential intrapopulation variation (Vissoto et al., 2022), because data on the interactions of multiple individuals in a population were mostly gathered to describe the interactions among species. In addition, studying individual variations in interaction with seed dispersers promotes the quantification of intraspecific variation in individual specialization and degree, which may provide insights into the ecological consequences of the relationship between individual variations and network metrics (Guerra et al., 2017; Carreira et al., 2020). Therefore, for many plant species, especially endangered species, it is necessary to focus on the functional traits of the partners interacting with them and the structural characteristics of the interaction network to better understand the interaction process and employ better management strategies. However, the structure of these individual-based networks and their driving factors are poorly understood (Friedemann et al., 2022).

Bird and plant traits are the most important factors affecting the network structure of seed dispersal and plant recruitment (Schupp et al., 2017; Li et al., 2019b; Li et al., 2022; Xiao, 2020). Among bird traits, body size plays a vital role in the seed dispersal network (Muñoz et al., 2017; Schupp et al., 2017), whereby large-bodied bird species are more important than small-bodied species because they serve as module hubs, connectors, or super-generalists (Olesen et al., 2007; Carreira et al., 2020). However, the role of other traits remains poorly explored (Li et al., 2019a; Li et al., 2019b; Schupp et al., 2019). In addition, some studies have suggested that bird foraging types play important roles in plant recruitment (Snow and Snow, 1988; Schupp et al., 2017); nevertheless, their role in the network has been ignored. Building networks with different foraging types could reveal an effective dispersal network related to plant recruitment, thereby providing evidence of plant persistence in fragmented forests. Moreover, the core plant species in the network are highlighted by food abundance and coverage traits (Cody, 1985). Fruit abundance is the most important factor affecting bird foraging behavior under disturbance (Cousens et al., 2010; Schupp et al., 2017). Trees with a high fruit abundance are always recognized as network module hubs (Guerra et al., 2017; Miguel et al., 2018), and they possess a high potential recruitment ability because of the increased seed removal (Cousens et al., 2010). Therefore, species traits are characterized as indicators for measuring the vital role of species in seed dispersal networks and plant recruitment in fragmented forests. Although several studies have reported a trait approach for understanding the role of species traits in the structure of seed dispersal networks at the community level (Guerra et al., 2017; Miguel et al., 2018; Crestani et al., 2019), the effects of species traits on network metrics within populations at the individual scale is poorly understood (but see: [Vissoto et al., 2022] from plant traits).

In the seed dispersal network, species diversity, abundance, and functional traits may affect dispersal distance (Gu et al., 2021; Zeng et al., 2021; Chen et al., 2022). Among bird traits, body size is the most important indicator for measuring dispersal distance (Sasal and Morales, 2013; Schupp et al., 2017). Large-bodied bird species play an important role in subsequent plant species recruitment because they provide plants with a longer dispersal distance than small-bodied species (Sasal and Morales, 2013; Rehm et al., 2019). Plant size also plays an important role in dispersal distance (Li et al., 2019b; Vissoto et al., 2022). Large, high trees provide a safe shelter for birds (Cody, 1985); thus, birds have a higher visiting frequency and a longer dispersal distance with large trees than with small trees (Schleuning et al., 2011; Vissoto et al., 2022). However, the effects of other species’ traits on the seed dispersal distance mediated by birds remain poorly elucidated. Therefore, advancing studies on the specific traits influencing individual seed dispersal networks and dispersal distance are considered key steps toward understanding plant persistence in fragmented forests.

*Taxus chinensis*, an endemic and relic tree species in China, has been listed as an endangered species by the International Union for Conservation of Nature (IUCN). *T*. *chinensis* is the dominant species in the study site, and its seeds are mainly dispersed by birds (Li et al., 2019a; Li et al., 2019b; Wang et al., 2022), rendering it an ideal model species to test the effect of individual traits and dispersal ability on interactions with seed dispersal agents. Given that this plant grows slowly and reproduces poorly in natural conditions, understanding the process underlying its seed dispersal may provide information for management strategies aiming to promote its spread (Li et al., 2019a; Li et al., 2019b; Li et al., 2022; Wang et al., 2022).

In this study, four individual-based networks were built for the endangered Chinese yew, *T. chinensis*, in a fragmented forest. The first two networks were built based on bird foraging type (swallowing and pecking networks), whereas the other two were built based on habitat type (networks in a bamboo patch and an evergreen broad-leaved forest patch). Two species-level network metrics (species degree and specialization, *d*’) were assessed among the four networks. With these results, the effect of species traits (bird and plant traits) on species-level network metrics and dispersal distance for *T. chinensis* was evaluated. This study aimed to address the following questions: (1) how do bird foraging type and habitat type affect the structure of individual-based dispersal networks? (2) How do species traits affect the species-level metrics of individual-based networks for the endangered trees? (3) How do species traits and species-level metrics affect seed dispersal distances? Our results will help guide the conservation and management of individual endangered trees in increasingly disturbed ecosystems, and provide new insights into individual-based seed dispersal mutualistic networks of endangered plant species under habitat fragmentation.

## Materials and methods

### Study site and species

**Figure 1:**
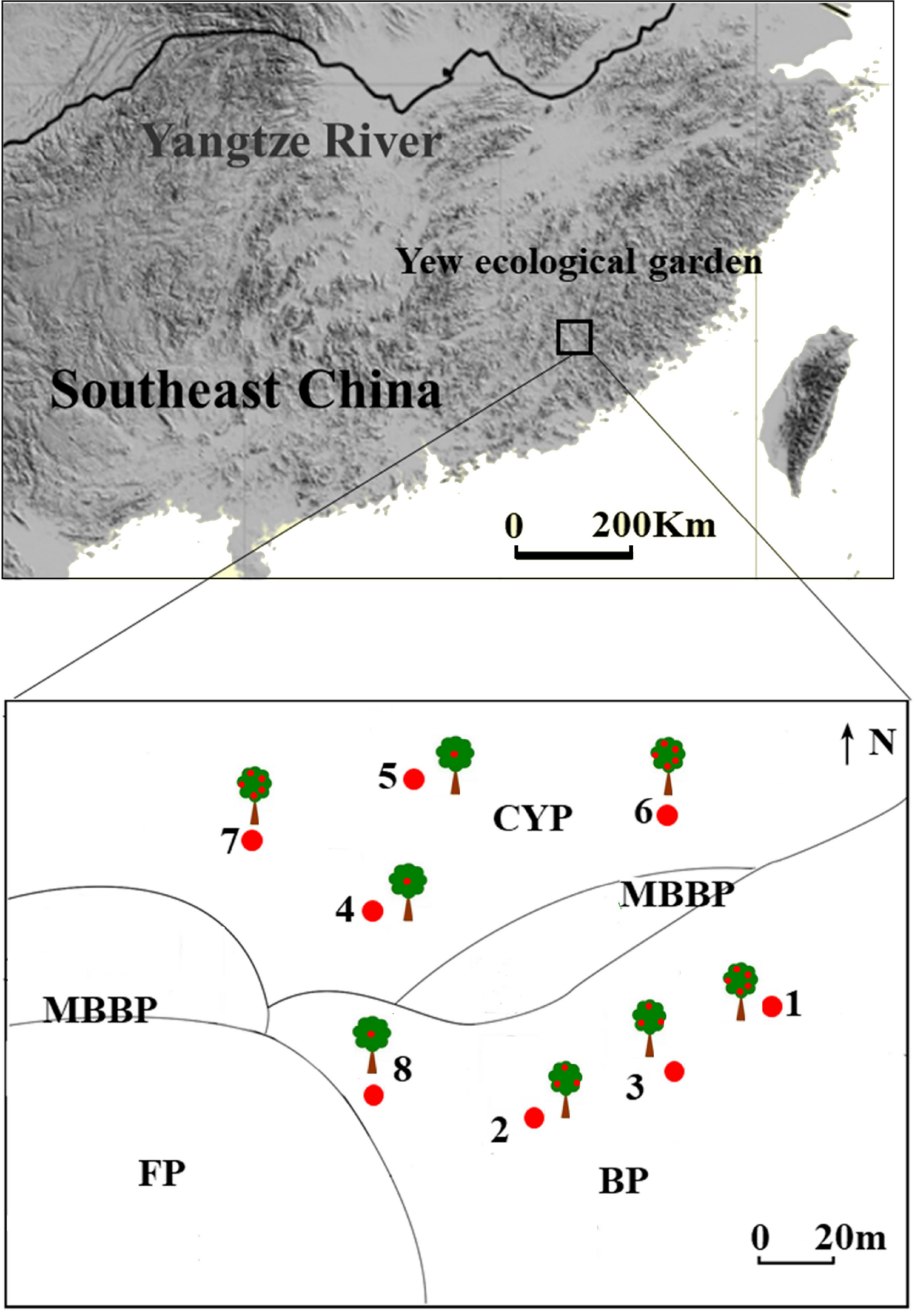
Study site and distribution of mother tree in different patches in yew ecological garden, Fujian province, southeast China. CYP, Evergreen broad-leaved forest patch; MBBP, Mixed bamboo and broad-leaved patch; BP, Bamboo patch; FP, Farmland patch. Red dots in the trees represent the fruit amount, 1 dot represent seeds < 15,000; 3 dots represent 15,000 < seeds < 20,000; 5 dots represent seeds > 20,000.

The study was conducted in a yew ecological garden (25° 15′–25° 35′ N; 116° 45′–116° 57′ E; elevation 895–1218 m a.s.l., slope gradient 27°) in the southern experimental area of the Meihua Mountain National Nature Reserve, west of Fujian province, southeast China. The Meihua Mountain National Nature Reserve is located on the southern edge of the subtropical zone, the transition zone between the subtropical and tropical zones. The annual average temperature, precipitation, and mean relative humidity are 13–18 °C, 1700–2000 mm, and 70–90%, respectively. This site has the largest natural population of *T. chinensis* in China (approximately 490 adults), including 200 trees older than 500 years. To protect these endangered trees, a national forest garden was established by the government in 2003; owing to their long-term human use, the vegetation around the forest garden is highly fragmented. The most important tree species in the remnant evergreen broad-leaved forest is *T. chinensis* (**Figure 1**).

*Taxus chinensis* is a natural and rare anti-cancer plant recognized as endangered worldwide (Thomas et al., 2013) and was declared a first-class national protected species by the Chinese government in 2003. This plant is an ancient relic tree species from the Quaternary glaciers and has existed on earth for 2.5 million years. Its fruiting season is late October to early December when it produces fleshy red arils containing a single seed (average diameter of 5 mm); an average tree bears more than 4,000 arils annually (Li et al., 2015; Li et al., 2019b).

### Study design

Fieldwork was performed between late October and early December in 2018 and 2019. We selected eight mature *T. chinensis* trees for study based on their large crop size and high visibility. Four individual trees were distributed in a bamboo forest and the others in an evergreen broad-leaved forest. Every individual tree was considered as an independent replicate sample because the distance between trees was over 30 m (**Figure 1**). Observation points were set on the slopes of the opposite mountain, and bird foraging behavior was studied using a telescope (Leica 70, Leica Microsystems GMBH, Mannheim, Germany). Each observation began with a bird visit to a mother tree and ended with its departure from the focal trees. Observations lasted 8 h daily (0630–1130, 1430–1730) during the fruiting seasons, accounting for a total of 464 h from 2018 to 2019. During observations, we identified and counted frugivorous bird species and foraging types (swallowing, pecking, crushing, and dropping) and recorded the number of seeds removed. Only the swallowing and pecking of fruits were treated as seed dispersal events for the fruit–bird interactions (Simmons et al., 2018).

Each departure of bird species from a tree was tracked in sessions that ended once the visual contact was lost or when the focal bird could no longer be distinguished from other conspecifics. In each session, the perching position (after leaving the study trees) of the bird was recorded every 30 s to note nearby characteristic landmarks for later mapping of the distance estimates. We assumed that the position of the first perch was a sufficient proxy for evaluating seed dispersal (Breitbach et al., 2010). Given that seeds are excreted through the digestive tract after swallowing, and discarded near parent trees after pecking, the seeds swallowed by birds spread farther than those that were pecked. According to the density-dependent hypothesis [Janzen-Connell hypothesis (Janzen, 1970)], seeds farther from their parent trees are more likely to germinate and establish seedlings. Therefore, we defined fruit-swallowing and fruit-pecking birds as those with a high and low seed dispersal contribution, respectively (Snow and Snow, 1988; Schupp et al., 2017; Simmons et al., 2018).

### Species traits

Three morphological bird traits (bill length, body weight, and wing length) were selected for their proven positively significant correlations with bird foraging types, seed dispersal distance, and visit frequency (Li et al., 2019b). Body weight was obtained from 10 individuals of each species in the zoological garden of Fujian and measured using an electronic scale. To obtain the bill and wing length, we measured five males and five females of each bird species using specimens preserved in the Museum of Nanjing Xiaozhuang University, and the mean value of each trait was used for analyses.

Three phenotypic traits (crop size, tree height, and tree cover) of individual mature trees were measured. Crop size was calculated by counting the total number of fruits in 10 randomly selected infructescences and extrapolating the sum to the remaining infructescences of each plant at the beginning of the fruiting period (when all fruits are formed). Tree height and cover were measured using the methods and protocols for plant community inventories (Fang et al., 2009).

### Data analysis

We constructed quantitative fruit–bird interaction networks using the bipartite R package (Dormann et al., 2009; R Core Team, 2020). Each individual-based fruit–bird interaction network was built using an adjacency matrix *A*, where *a*_ij_ = the number of interactions from an individual fruit (*j*) by the bird species (*i*) or zero. For each tree, fruit–bird interaction networks were built according to the habitat (bamboo and evergreen broad-leaved forest patches) and foraging types (swallowing and pecking), totaling four interaction networks.

We analyzed plant-bird associations at both the species level (based on networks for individual plant or bird species interacting with partners) and the network level (based on networks at the whole-network level). For the species level, the standardized Kullback–Leibler distance (the index *d’*, specialization) was used to calculate the degree of interaction specialization per bird species (or plant individual), which considers the proportional utilization and availability of interaction partners and therefore provides a robust estimate of specialization at the species level (Bluthgen et al., 2006). We then calculated species degree (degree), which is the number of species associated with other related species in the network (Bascompte et al., 2006; Bascompte and Jordano, 2007).

For the network level, we analyzed four network metrics including connectance (*C*), the proportion of realized/possible links in a network (Dunne et al., 2002); specialization (*H*_2_), the standardized two-dimensional Shannon entropy, ranging between 0 and 1.0 for extreme generalization and specialization, respectively (Bluthgen et al., 2006); nestedness [weighted nested overlap and decreasing fill (NODF)], which describes the tendency for specialist nodes of one type to interact with generalist nodes of the other type, such that more specialist nodes interact with a subset of the nodes that are more connected with generalized nodes (Almeida-Neto et al., 2008; Montesinos-Navarro et al., 2017); and modularity (*Q*), which quantifies whether interactions in each patch formed distinct modules. The DIRTLPAwb + algorithm was used for maximizing modularity (Beckett, 2016) to identify groups of seed–birds interactions that were stronger within, rather than among, modules.

To test the role of species traits in the seed dispersal networks, the random forest (RF) algorithm was used to plot the partial effects of bird traits and study years on the two species-level network metrics (degree, specialization) (R package randomForest); the RF algorithm was also used to test the effects of plant traits on the species-level network metrics. To test the role of species traits in seed dispersal distance, the RF algorithm was used to plot the partial effects of bird traits, two network metrics, and study years on the dispersal distance; the RF algorithm was also used to test the effects of plant traits on their dispersal distance.

## Results

### Structure of individual-based dispersal network

**Figure 2:**
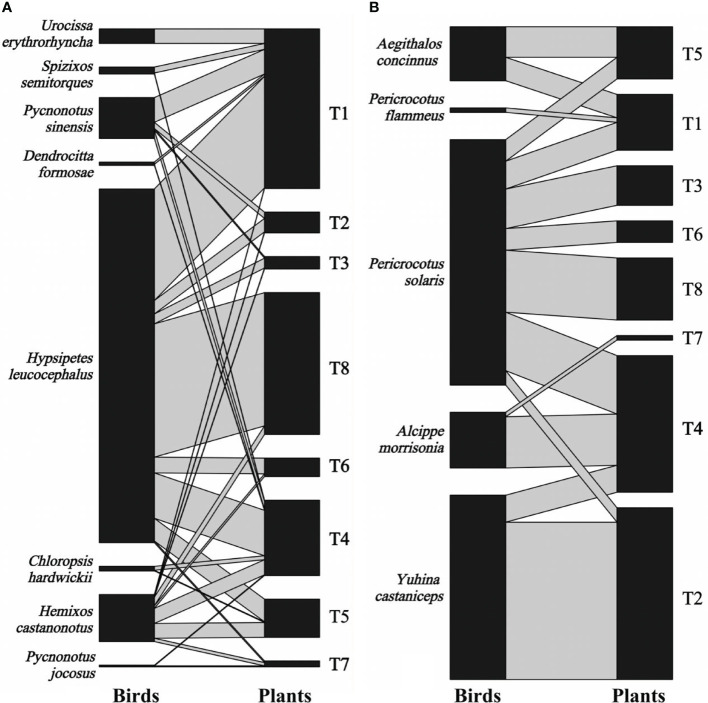
Bipartite graph of fruit-bird interaction networks based on bird swallowing **(A)** and pecking behavior **(B)** in the yew ecological garden, Fujian province, southeast China from 2018 to 2019. The size of the left square (animals) and right square (plant individuals) indicated the relative abundance of the bird and fruit interaction, respectively. The grey line indicated the interaction between birds and fruits, and the line thickness indicated the interaction strength. T1-T8 indicated the tree numbers 1 to 8.

We recorded 2,041 visits by 13 bird species on eight individual *T. chinensis* across two forest patches for 2 years. Eight bird species foraged *T. chinensis* fruits by swallowing behavior, resulting in 1,436 visits, whereas the remaining five bird species foraged *T. chinensis* fruits by pecking behavior, resulting in 605 visits (**Figure 2**). Degree varied between 3 and 10 bird species per tree (5.38 ± 2.58; mean ± S.D., *n* = 16), whereas specialization ranged between 0.05 and 0.59 (0.21 ± 0.13, *n* = 16). Visitation rates ranged from 6 to 440 (127.56 ± 146.78, *n* = 16) visits per individual. *Hypsipetes leucocephalus* was the most frequent disperser (52.67%, *n* = 1075 visits), followed by *Pericrocotus solaris* (13.38%, *n* = 273), *Pericrocotus flammeus*, and *Pycnonotus jocosus* (0.02%, *n* = 5).

**Figure 3:**
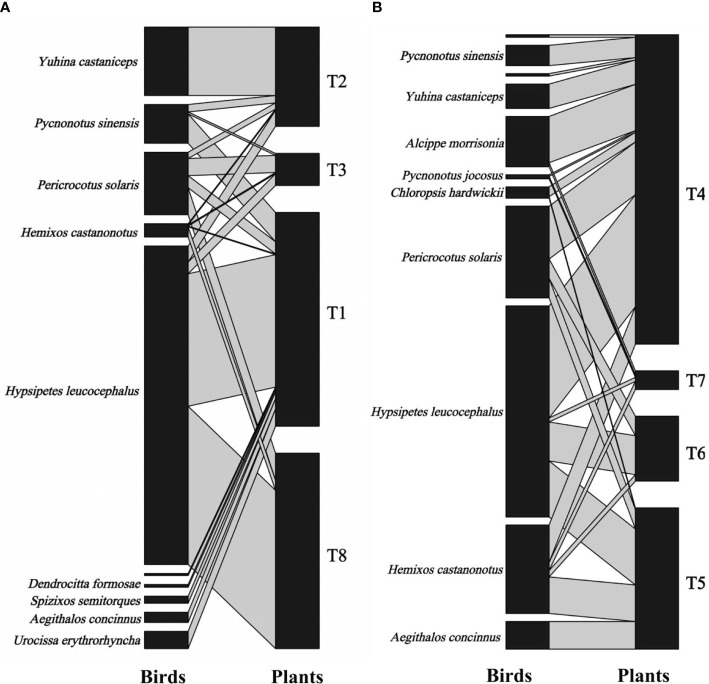
Bipartite graph of fruit-bird interaction networks in the bamboo patch **(A)** and the evergreen broad patch **(B)** in the yew ecological garden, Fujian province, southeast China from 2018 to 2019. The size of the square indicated the relative abundance of the bird and fruit interaction, respectively. The line thickness indicated the interaction strength.T1-T8 indicated the tree numbers 1 to 8.

The fruit–bird interaction networks exhibited intermediate nestedness (weighted NODF = 41.22) and connectance (*C* = 0.41) with low modularity (*M* = 0.28) (**Table 1**). Quantitative networks had significantly higher nestedness (Z-scores > 2, *P* < 0.01) and lower modularity (Z-scores < -2, *P* < 0.01) than those in most null models (**Table S1**). Compared to bird pecking networks, bird swallowing networks had higher nestedness and connectance and lower modules and specialization (*H_2_’*) (**Figure 2**; **Table 1**). Similarly, fruit–bird interaction networks in the evergreen broad-leaved patch had higher nestedness and connectance and lower modules and specialization (*H_2_’*) than those in the bamboo patch (**Figure 3**; **Table 1**).

**Table 1:** Comparison of network metrics among four seed dispersal networks based on bird foraging type (swallowing and pecking) and patch type (bamboo patch and evergreen broad-leaved forest patch) in the yew ecological garden representing a fragmented forest in Southeast China.

Descriptors	Connectance (*C*)	Specialization (*H*_2_ *’*)	Weighted NODF	Modularity (*Q)*
Swallowing networks	0.453	0.262	41.220	0.178
Pecking networks	0.350	0.598	25.000	0.469
Network in the bamboo patch	0.525	0.517	33.202	0.287
Network in the evergreen broad-leaved forest patch	0.500	0.212	53.087	0.190

### Effects of species traits on species-level metrics

**Figure 4:**
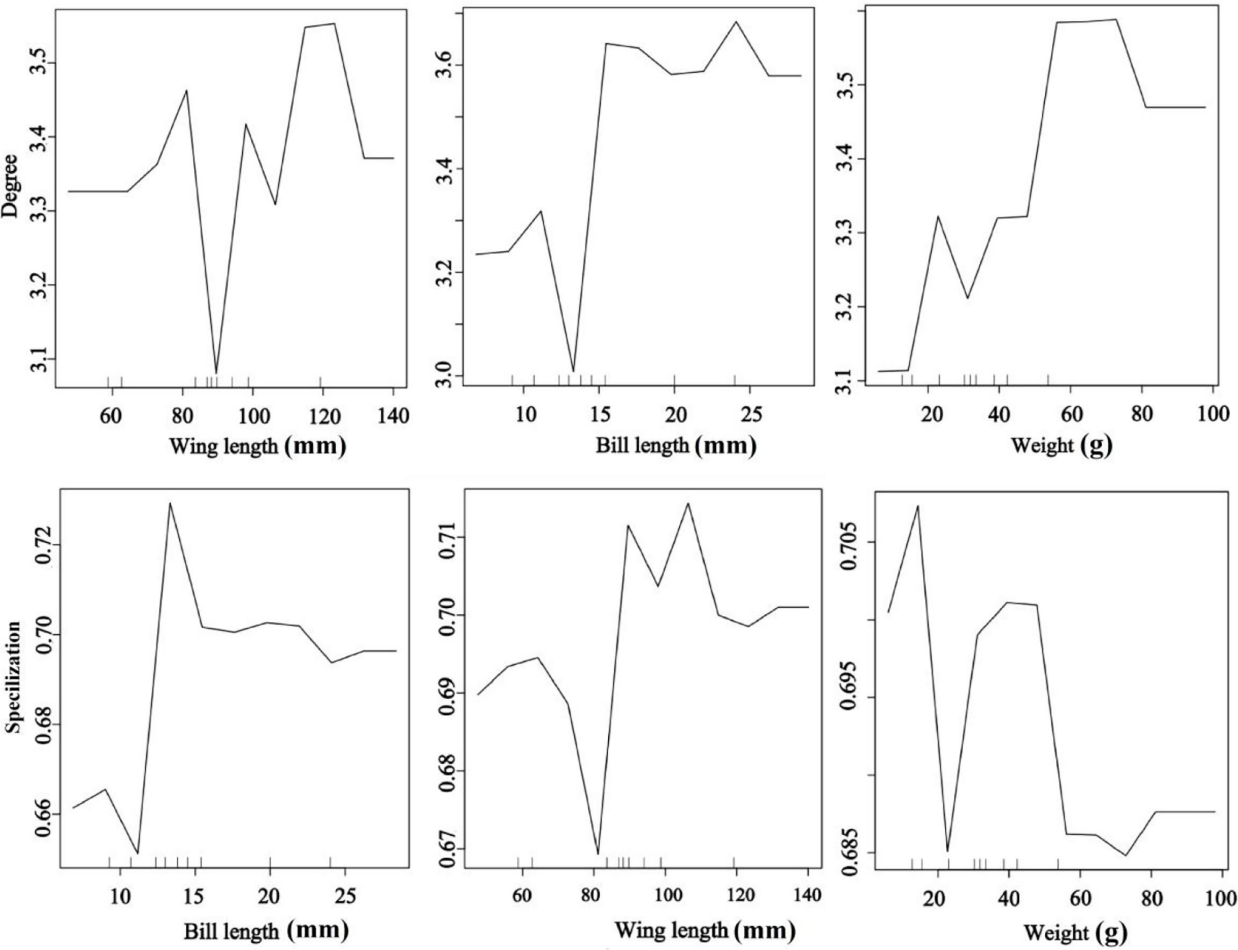
Effects of bird traits on the network metrics at the species level (degree and specialization) representing a fragmented forest in Southeast China. Results were determined using the Random Forest algorithm and showed the partial effects of independent variables on degree and specialization, respectively.

Bird morphological traits were important for seed dispersal networks (**Figure 4**). The RF results explored the important role of bird traits on species degree and specialization in the seed dispersal networks. The bird species with long wing and bill lengths and heavier weights had a higher species degree value than the smaller species. Medium bill and wing lengths and low weight were the most important traits for specialization (**Figure 4**). The results showed that large-bodied birds visited more individual trees, suggesting a higher contribution to seed dispersal of endangered *T. chinensis* trees (random forest: 78.15% of degree and specialization data could be explained by three variables).

**Figure 5:**
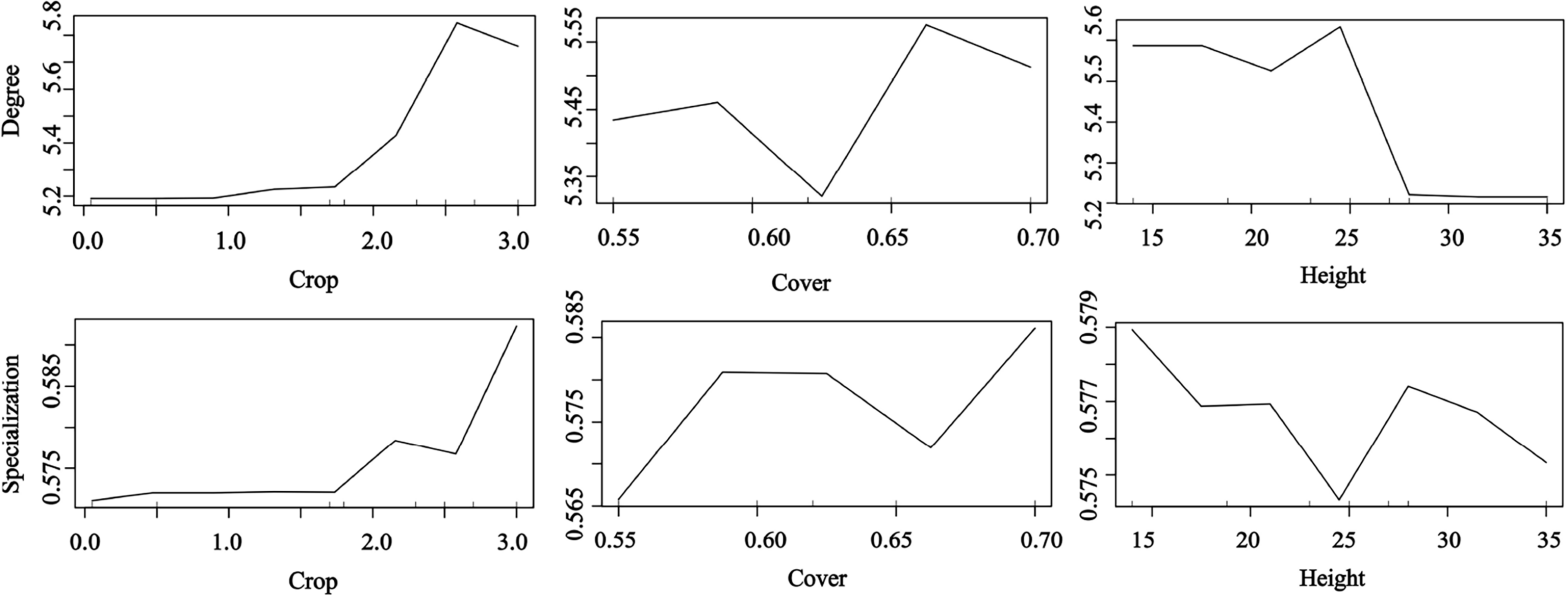
Effects of plant traits on the network metrics at the species level (degree and specialization) representing a fragmented forest in Southeast China. Results were determined using the Random Forest algorithm and showed the partial effects of independent variables on degree and specialization, respectively.

Our results confirm that plant traits contribute to seed dispersal networks (**Figure 5**). The RF results showed that the degree and specialization values in the networks were significantly and positively affected by fruit crop size but significantly and negatively affected by tree height. The specialization value increased with tree cover; trees with high and low cover had a higher contribution than those with medium cover (random forest: 69.23% of degree and specialization data could be explained by three variables) (**Figure 5**).

### Effects of species traits on seed dispersal distance

**Figure 6:**
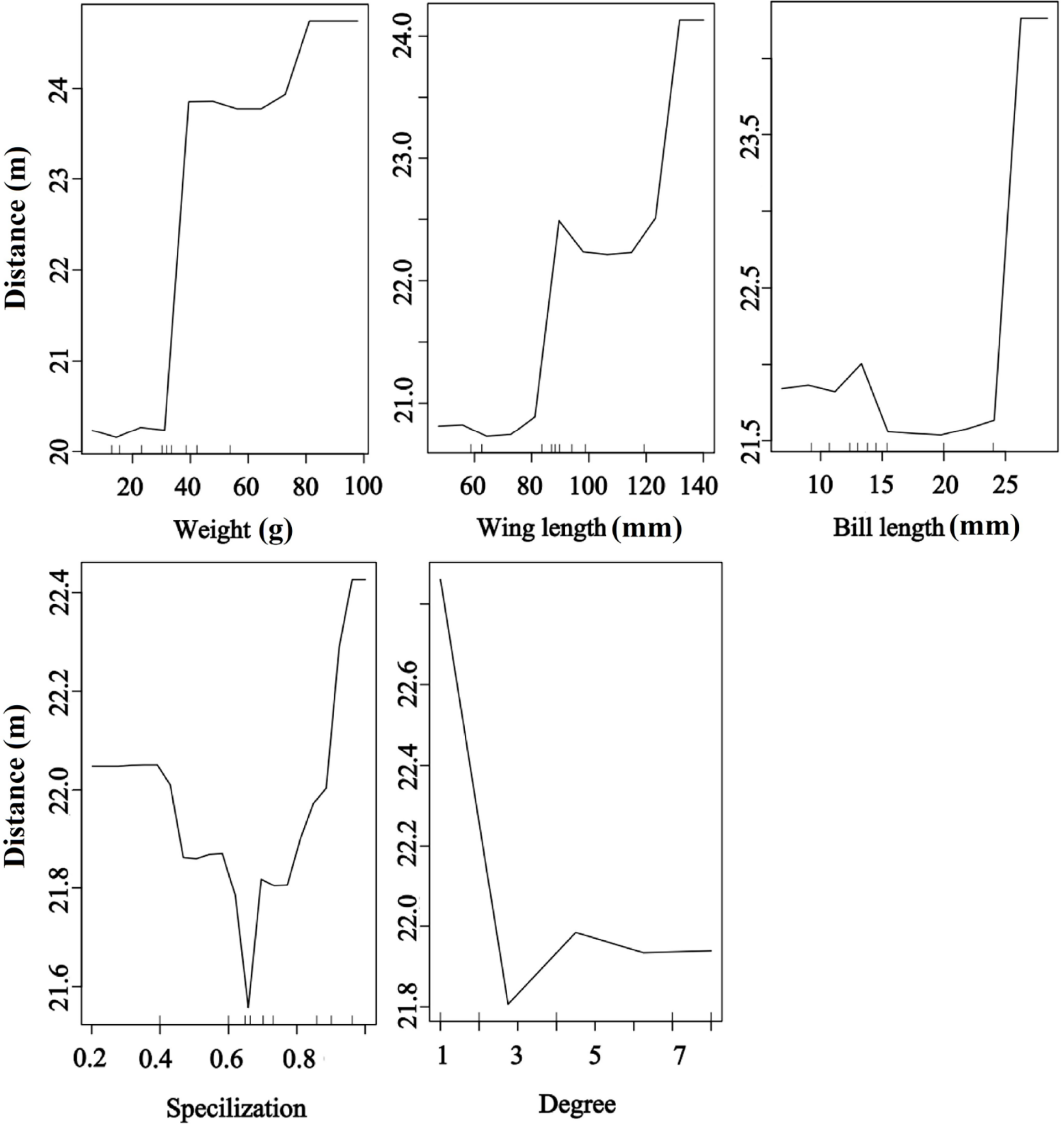
Effects of bird traits and two network metrics on the dispersal distance representing a fragmented forest in Southeast China. Results were determined using the Random Forest algorithm and showed the partial effects of independent variables on dispersal distance.

Bird morphological traits and species-level network metrics were important for seed dispersal distance. The RF results showed that seed dispersal distance was significantly and positively affected by bird morphological traits but significantly and negatively affected by degree, implying that large body-sized birds and less connected species provided a higher dispersal distance than small body-sized birds and more connected species. Moreover, the birds with higher specialization values contributed to higher dispersal distances than those with other values (random forest: 78.05% of distance data could be explained by five variables) (**Figure 6**).

**Figure 7:**
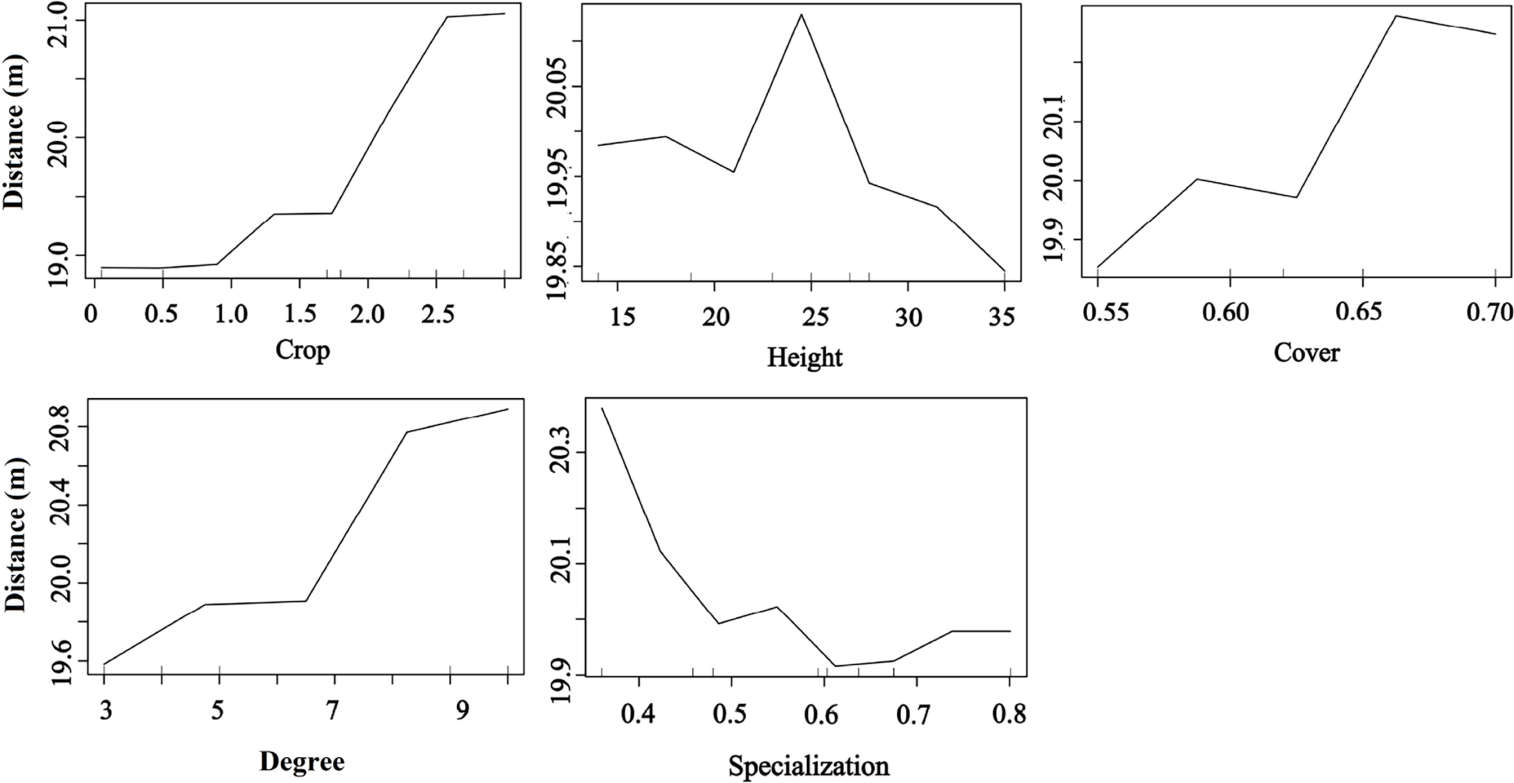
Effects of plant traits and two network metrics on the dispersal distance representing a fragmented forest in Southeast China. Results were determined using the Random Forest algorithm and showed the partial effects of independent variables on dispersal distance.

Plant phenotypic traits also played a vital role in seed dispersal distance. The RF results showed that the seed dispersal distance was positively affected by fruit crops, tree cover, and degree but decreased with the plant specialization value, indicating that the higher the tree crops, the larger the tree cover, and frequent bird species visits were beneficial for an increased seed dispersal distance. Moreover, trees with medium height had a higher dispersal distance than those with other heights (random forest: 83.12% of distance data could be explained by five variables) (**Figure 7**).

## Discussion

This study revealed that the interaction network between individual *T. chinensis* trees and their bird partners was influenced by bird foraging and habitat types. Compared to bird-pecking and bird–fruit networks in the bamboo patch habitat, bird-swallowing and bird–fruit networks in the evergreen broad-leaved patch habitat had higher nestedness and connectance but lower modules and specialization. These results indicated that bird traits such as body weight, wing and bill lengths, and plant traits such as height, crop size, and cover significantly affected species-level network metrics such as degree and specialization. Furthermore, seed dispersal distance was influenced by species traits and the species-level metrics of fruit–bird interaction networks. These results provide new insights into individual-based seed dispersal mutualistic networks of endangered plant species under habitat fragmentation.

Numerous studies have shown that nestedness and modularity are important structural metrics in ecological networks (Almeida-Neto et al., 2008; Fortuna et al., 2010). Nestedness is the tendency for specialists to interact with a proper subset of species interacting with more generalists (Bascompte and Jordano, 2007), whereas a module is a densely connected and non-overlapping subset of species (Olesen et al., 2007). Previous studies have shown that a highly connected and nested architecture stabilizes mutualistic communities but harms the stability of antagonistic networks (Thebault and Fontaine, 2010). In this study, compared to bird pecking networks, bird swallowing networks had higher nestedness and lower modules. Similarly, fruit–bird interaction networks in the evergreen broad-leaved patch had higher nestedness and lower modules than those in the bamboo patch. The nested structure suggests that the plant individuals in bird swallowing or evergreen broad-leaved patch networks interacted with a higher number of dispersers, whereas the other individuals interacted with a subset of seed dispersers of the more generalist individuals. Simmons et al. (2018) distinguished between bird swallowing and pecking owing to their opposing consequences for plant reproductive success and found that the removal of bird pecking interactions caused a significant increase in network-level metrics such as connectance and nestedness. In addition, the modular structure was detected when interaction frequencies were considered. This indicates a partition of seed dispersers among individuals within the population, where subsets of individuals interact strongly with distinct seed dispersers, forming interaction modules (Vázquez et al., 2007; Krishna et al., 2008). Furthermore, our results revealed that individuals in the bird-swallowing and the evergreen broad-leaved patch networks might acquire a higher persistence ability than the others, consistent with other studies; the nestedness and modularity of both networks affect the robustness of a network against species extinction (Bastolla et al., 2009; Tylianakis et al., 2010).

Our results highlighted the importance of species traits in the species-level metrics for the individual networks. For bird traits, a positive relationship was found between species degree and bird wing and bill lengths, and weight. Compared to small-bodied species, large-bodied species consumed more fruits, consequently increasing these species in the network (Schupp et al., 2017). Furthermore, medium-bodied species played an important role in the specialization of the networks. Specialization in fewer dispersers can be beneficial if dispersers exhibit preferences for occupying highly suitable sites for seed germination (Garrote et al., 2018), which are characteristic features of *T. chinensis* individuals. Additionally, interactions with multiple (more exclusive) disperser species and dispersal distance were associated with plant traits. The positive relationship between degree, specialization, and plant crop size was influenced by a few individuals with extremely high fruit availability, congruent with other contributions (Sargent, 1990; Gleditsch et al., 2017). This indicates that dispersal opportunities are increased for individuals in conditions with high fruit availability, suggesting the existence of potential facilitation among plants that share seed dispersers (facilitation hypothesis; [Gleditsch et al., 2017]). The trees with high and low cover had a higher contribution to specialization than the others; this could be attributed to the safety requirements of birds. Trees with high cover provide better shelter for birds, whereas birds living in trees with low cover are easily preyed on (Cody, 1985).

This study established that large-bodied species supplied a longer dispersal distance than small-bodied species, consistent with previous studies (Chen and Moles, 2015; Muñoz et al., 2017). Most importantly, we identified that long dispersal distance is related to network structure. Low connectance and highly specialized species provided longer dispersal distances than others; consequently, an increasing extinction risk was observed in the networks, owing to the long distance generated by these redundant species (Garrote et al., 2018). Furthermore, plant phenotypic traits played a vital role in seed dispersal distance. Our results showed that the seed dispersal distance was positively associated with crop size, cover, and degree but was negatively associated with the plant specialization value. This indicated that trees with high fruit density and cover could acquire a longer dispersal distance than others, enhancing their persistence in the fragmented forest, congruent with contributions of the degree value of plant species in a previous study (Vissoto et al., 2022).

Our study demonstrated the effect of species traits on bird species–*T. chinensis* interactions from a bird (swallowing and pecking networks) and plant perspective (networks in a bamboo patch and an evergreen broad-leaved forest patch). Additionally, the importance of bird traits and their network metrics in the seed dispersal distance (as an outcome for plant recruitment) was revealed. Seed recruitment is an important indicator for assessing the specific traits of a network (Schupp et al., 2017; Rehm et al., 2019); however, this aspect was not considered in this study. Future studies should focus on the relationship between species traits and the processes of seed recruitment, which could not only elucidate the relationship between bird and plant traits but also explore how much each trait contributes to seed dispersal networks and plant recruitment. In our study, we developed the seed dispersal network at an individual scale, which could help to explore the persistence of endangered tree species. Future studies should build the network at a community scale to explore the role of endangered species in the network and the maintenance of biodiversity. Overall, this study fills important gaps in the identification of the effect of species traits on individual-based mutualistic networks. Furthermore, it has relevant implications for conserving and managing individual endangered trees in increasingly disturbed ecosystems.

## Data availability statement

The original contributions presented in the study are included in the article/**Supplementary Material**. Further inquiries can be directed to the corresponding author.

## Author contributions

NL and ZW conceived and designed the experiments. NL and XY analyzed the data and wrote the paper. All authors performed the experiments and read and approved the final manuscript.

## Funding

This study was supported by the National Natural Science Foundation of China (Grant No. 32171528, 31700468), the Natural Science Foundation of Jiangsu Province (Grant No. BK2017636, BK20221180) and the Key Subject of Ecology of Jiangsu Province (SUJIAOYANHAN〔2022〕No.2).

## Acknowledgments

We thank Shuai Zhang for their contributions in the field, and anonymous reviewers for valuable comments. We thank Editage for its linguistic assistance during the preparation of this manuscript.

## Conflict of interest

The authors declare that the research was conducted in the absence of any commercial or financial relationships that could be construed as a potential conflict of interest.

## Publisher’s note

All claims expressed in this article are solely those of the authors and do not necessarily represent those of their affiliated organizations, or those of the publisher, the editors and the reviewers. Any product that may be evaluated in this article, or claim that may be made by its manufacturer, is not guaranteed or endorsed by the publisher.
