# Supplementary material for: Importance of species traits on individual-based seed dispersal networks and dispersal distance for endangered trees in a fragmented forest
Source: Front Plant Sci. 2022 Sep 21;13:1010352. doi: 10.3389/fpls.2022.1010352 (PMC9534520; doi:10.3389/fpls.2022.1010352)
Supplement: Supplementary file 1 [file Table_1.docx]

**Table S1** Results from *r2dtable* null model testing the significance of the metrics calculated for the fruit-bird interaction networks (Dormann *et al.*, 2008). For each null model, we generated a set of 1000 randomized networks, and compared the distribution of network indices to the observed value. *r2dtable* null model generates a set of random 2-way tables with given marginal totals using Patefield’s algorithm (Yodzis, 1981).

| Descriptors | Weighted NODF | | | Modularity | | |
| --- | --- | --- | --- | --- | --- | --- |
|  | Observed value | Mean of the null model | Z-score | Observed value | Mean of the null model | Z-score |
| Swallowing networks | 41.220 | 69.849 | 27.564 | 0.178 | 0.028 | -4.985 |
| Pecking networks | 25.000 | 56.787 | 18.386 | 0.469 | 0.051 | -4.509 |
| Networks in the bamboo patch | 33.203 | 39.820 | 29.549 | 0.288 | 0.030 | -4.195 |
| Networks in the evergreen broad-leaved forest patch | 53.087 | 57.810 | 44.730 | 0.190 | 0.047 | -4.402 |
